# Public Health in the Age of Health Care Reform

**DOI:** 10.5888/pcd9.120151

**Published:** 2012-10-11

**Authors:** Catherine B. Kemp

Preventive medicine has vast unused possibilities for improving the health of the population, partly in ways that require compulsory controls, like quarantine and compulsory immunization, partly through systematic arrangements for personal check-ups and early diagnosis and treatment. Without going into the controversial question of socialized medicine, it is clear that health is a national asset, and the undisputed judgment of the people is that it is worth conserving, more or less regardless of whether it can be done on purely commercial principles. JM Clark, 1950 (1)

One might assume that the foregoing quotation is from recent debates on the Patient Protection and Affordable Care Act (PPACA) (2); however, it actually appeared in an article written more than 60 years ago. The author, an economist, was articulating his perspective on the effect of President Franklin Roosevelt’s New Deal and the influences of Keynesian economics. His remarks illustrate that issues related to health care reform — now the PPACA — are long-standing. Discussions of health care reform have been a major part of American public discourse in nearly every decade since President Theodore Roosevelt’s 1912 platform for re-election included a proposal for universal health insurance (3). Historically and currently, the health care debate has emphasized the treatment of people once they become ill. This emphasis ignores the basic fact that maintaining the health of the population is essential to our economy, our national security, and our success as a nation. The benefits of a healthy population extend far beyond health care costs. Although treating illness is an indispensable part of health care, reforming and strengthening our public health system are equally important to improving the health of individuals and of our population as a whole and to managing finite resources effectively.

To define “public health,” many turn to Dr. C.E.A. Winslow, a pioneer instrumental in the development of this discipline, who described it as: the science and the art of preventing disease, prolonging life, and promoting physical health and efficiency through organized community efforts for the sanitation of the environment, the control of community infections, the education of the individual in principles of personal hygiene, the organization of medical and nursing service for the early diagnosis and preventive treatment of disease, and the development of the social machinery which will ensure to every individual in the community a standard of living adequate for the maintenance of health (4).

Indeed, the Partnership for Prevention defines the 3 main goals of public health as protection of the population’s health and safety, promotion of improved health for everyone, and prevention of the consequences and suffering resulting from disease (5).

Although there is some overlap, the discipline of public health differs from health care practice, which involves assessing health, diagnosing symptoms, treating diseases, and managing chronic impairments. Public health’s primary function is to protect and promote the population’s health through prevention, environmental protection, and public regulations and policies. Kenneth Arrow made this very point in a 1963 essay (6) when he described how medical care is but 1 aspect of health care and that other aspects such as sanitation, shelter, nutrition, and clothing are perhaps even more important to health, especially when a person lacks them (6). Discussions about disease prevention and health promotion today are too frequently overshadowed by politics and debates about medical interventions and technologies. Meanwhile, the American health care system is expensive; its costs are rising and consistently outpacing inflation, from 5.2% of gross domestic product in 1960 to 17.6% in 2009,a trajectory that is expected to persist (7).

Our population is aging. Projected demographic shifts over the next 20 years underscore the need to incorporate effective preventive health services into public health. The US Department of Health and Human Services indicates that in 2009, 39.6 million Americans were aged 65 years or older and accounted for 12.9% of the total population. By 2030, this number is expected to increase to 72.1 million, or 19.7% (8). Similarly, the Centers for Disease Control and Prevention projects the US population to grow by 29.2% by 2030 and the segment aged 65 and older to increase by 104.2% (9). Other government data predict that the proportion of older Americans will more than double during the next several decades, while the segment aged 20 to 64 will shrink by nearly 5% (10). These trends will translate into fewer wage-earners paying taxes to fund Medicare for the rapidly increasing number of retirees.

These demographic shifts highlight the need to prevent or delay the onset of age-related illnesses such as cancer, diabetes, and heart disease. These 3 diseases are major drivers of health care expenditures and are associated with modifiable risks that public health interventions can influence (11). Perrott points to the significance of this evolution: “Among the various characteristics of recent population trends, aging of the population is one of the most fundamental in its bearing on national health” (12).

Gostin and colleagues present evidence that issues related to socioeconomics, behavior, and the environment reduce illness and death by a factor of 4 to 1, compared with the effect of clinical care (13). Historically, public health has been at the forefront of US health care through sanitation and disease prevention. Medical advances of the late 20th and early 21st centuries have shifted the focus of health care to diagnosis and treatment, eclipsing public health’s important role in preventing disease. Gostin and colleagues (13) argue that the reintegration of public health and health care would increase efficiencies, reduce costs, and improve health outcomes.

A 2009 report (14) on the role of local health departments (LHDs) contends that health care reform emphasizes the vital role of public health. The report illustrates the contradiction between expanding health care insurance coverage while reducing resources for LHDs and presents data to illustrate that health care accounts for only 10% of health outcomes; the remaining 90% are influenced by a complex mix of determinants of health including behaviors, socioeconomics, and physical environments:

While medical care can prolong survival and improve prognosis after some serious diseases, more important for the health of the population as a whole are the social and economic conditions that make people ill and in need of medical care in the first place. Poor social and economic circumstances affect health throughout life. People further down the social ladder usually run at least twice the risk of serious illness and premature death as those near the top (14).

The report asserts that a consensus is possible on how to target these underlying social and economic issues and maintains that increasing funding of LHDs could potentially save $20 in health care costs for every prevention dollar spent; this can be achieved by focusing on the principal causes of poor health, such as smoking, poverty, homelessness, pollution, substance abuse, and violence (14).

Jacobson and Gostin also call for adequate, secure, long-term funding for public health: “The public health system is badly frayed and needs to be rebuilt structurally and redesigned conceptually” (15). Meyer et al (16) emphasize the importance of health promotion and disease prevention and maintain that, contrary to conventional wisdom, lack of access to health care *is not* the principal driver of illness and death. They agree that the causes of disease involve behaviors, the environment, and socioeconomics. Furthermore, they contend that the principal objective of a health system should be improving the population’s health, which does not necessarily result from medical interventions. They point out that evidence demonstrating the cost-effectiveness of public health interventions is abundant, whereas the same cannot be said about our expensive health care system.

If public health is to lead the cause of improving the population’s health and reducing the need for expensive medical care, we must recognize that adequate and secure funding for public health is cost-effective not only in terms of health care, but in terms of the national economy. We also must address the problem of lack of uniformity among local, state, and national public health agencies: “The nation’s state health departments vary widely in their scope of responsibilities and their size. . . . In some states, responsibility for local health department activities is centralized in the state agency; in other states the local health responsibilities are shared between state and local entities; and in still other states, the local health agency structure is almost entirely decentralized” (5).

Several areas of the PPACA attempt to address public health issues, such as mandating that all insurance policies provide coverage without copayments for a range of preventive services and screenings. Details on which screenings will be covered and how they will be delivered and paid for are not adequately described. Whereas the PPACA designates some funding to subsidize state Medicaid costs for preventive services and screenings, the funding is temporary, and presumably the burden will shift to state budgets after PPACA funding expires. The legislation also establishes the Preventive Services Task Force, whose duties will range from developing topic areas and recommendations to assisting the federal government in improving integration of health objectives into targeted settings. In addition, the legislation creates some direct services, such as grants to fund public school-based health centers serving large populations of children who are covered by state Medicaid or Social Security programs.

The federal government has also increased its focus on public health in *Healthy People 2020* by defining a set of objectives that highlight America’s high-priority health issues (17). These objectives, or “leading health indicators,” developed with input from the Institute of Medicine, take into account that both risk factors and health determinants vary during the life span, affecting states of health and development of diseases (18). These indicators include access to care, healthy behaviors, chronic disease, environmental determinants, social determinants, injury, mental health, maternal and infant health, responsible sexual behavior, substance abuse, tobacco, and quality of care.

These examples highlight federal efforts to address public health concerns. However, individually or collectively, they do not have the same potential to improve the population’s health as public health does, given adequate resources. The PPACA’s main focus is on health insurance and includes only a few provisions that address public health. Resolving the complex challenges that lay ahead as our population ages, as demand for services increases, and resources dwindle will require a multifaceted approach. Public health has the greatest potential for protecting the public’s health and reducing demand for expensive health care products and services.

Public health has been the driving force in the United States in creating the infrastructure and programs for protecting the population’s well-being. Recently, it has been overshadowed by new technologies that diagnose and treat illness instead of preventing it. This shift has resulted in rapid growth of health care expenditures, which have consistently outpaced the rate of inflation. This trend is projected to continue. The demand for publicly funded health care will continue to grow as Medicare rolls swell with aging Americans and as Medicaid coverage expands under new reforms. These opposing forces will collide unless something is done to alter their course. The best and most cost-effective way to manage these pressures and to protect our nation’s health is to reform the infrastructure and ensure adequate funding of public health.
